# Middle Pancreatectomy for Traumatic Main Pancreatic Duct Injury with Delayed Presentation: Two Case Series

**DOI:** 10.70352/scrj.cr.25-0094

**Published:** 2025-06-17

**Authors:** Yuki Itagaki, Shintaro Takeuchi, Takehiro Noji, Yuma Ebihara, Masataka Wada, Kimitaka Tanaka, Aya Matsui, Yoshitsugu Nakanishi, Toshimichi Asano, Toru Nakamura, Satoshi Hirano

**Affiliations:** Department of Gastroenterological Surgery II, Hokkaido University Faculty of Medicine, Sapporo, Hokkaido, Japan

**Keywords:** trauma, pancreatic injury, pancreatic trauma, main pancreatic duct injury, middle pancreatectomy, central pancreatectomy, endoscopic retrograde pancreatography, delayed presentation

## Abstract

**INTRODUCTION:**

Pancreatic trauma is an uncommon, yet potentially lethal condition, with main pancreatic duct (MPD) disruption guiding surgical management. Middle pancreatectomy (MP) with Roux-en-Y pancreatojejunostomy (PJ) offers an organ-preserving alternative to distal pancreatectomy, particularly for young patients. However, the extent of its applicability and the specific surgical techniques—including key technical tips—remain unclear in the context of traumatic pancreatic injury. This is especially true in cases of delayed presentation, where severe intra-abdominal inflammation further complicates surgical intervention.

**CASE PRESENTATION:**

We report 2 cases of young patients with MPD injuries from blunt trauma, both presenting late with significant peripancreatic contamination. Case 1 included a 22-year-old male who sustained pancreatic and liver injuries while skiing. He was transferred 30 hours post-injury with stable hemodynamics. Endoscopic retrograde pancreatography (ERP) confirmed MPD disruption. Intraoperatively, saponification obscured the anatomical structures, but MP with PJ was successfully performed. The patient recovered without major complications. Case 2 involved a 17-year-old female who was initially observed at another hospital after a traffic accident. Three days later, she developed peritonitis, and a retrospective computed tomography review revealed a pancreatic body rupture. An ERP confirmed MPD disruption. During surgery, extensive inflammation and adhesions were noted, and the MPD was extremely small. Despite technical complexities, an MP with PJ was successfully completed. The pancreatic fistula from the pancreatic head stump required drainage treatment following spinal surgery for vertebral fractures, and the patient recovered without sequelae.

**CONCLUSIONS:**

MP with Roux-en-Y PJ is a technically challenging but viable approach for MPD injuries in young patients, even with delayed presentation. It preserves the pancreatic and splenic functions, making it a valuable approach for young patients when performed by experienced surgeons. These cases demonstrate the clinical impact and potential implications of MP as a viable treatment approach for pancreatic trauma.

## Abbreviations

CRP
: C-reactive protein
CT
: computed tomography
DP
: distal pancreatectomy
ERP
: endoscopic retrograde pancreatography
MP
: middle pancreatectomy
MPD
: main pancreatic duct
PF
: pancreatic fistula
PJ
: pancreatojejunostomy

## INTRODUCTION

Pancreatic trauma is a rare but life-threatening condition, accounting for 0.2%–0.6% of all trauma.^1–3)^ In particular, MPD disruption is a lethal situation and a critical determinant in the surgical management of blunt pancreatic injury.^2,4–7)^ Pancreatic trauma typically occurs in a normal pancreas, which is inherently more prone to PF formation due to its soft and fragile tissue.^8)^ Additionally, traumatic injury itself is an independent risk factor for developing a PF following pancreatectomy.^9)^ This risk is further exacerbated when there is a prolonged interval between the injury and presentation, leading to inflammation and autodigestion of the pancreas and surrounding tissues, which can compromise the success of organ-preserving surgery.^10)^

MP with Roux-en Y PJ for traumatic pancreatic injury, 1st reported by Letton and Wilson, is a commonly performed procedure for pancreatic neck and body trauma.^2,11,12)^ Since this organ-sparing technique preserves both the pancreatic glandular tissue and the spleen, it is particularly suitable for pediatric and young patients whenever feasible.^13)^ Its application aims to reduce the risk of future diabetes, body weight loss, and immune dysfunction, thereby contributing to better long-term outcomes.^14,15)^ Despite its function-preserving advantages, this procedure is not recommended in Western guidelines due to its time-consuming nature and technical difficulty.^16,17)^ Instead, DP with splenectomy is considered the gold standard and remains the most commonly performed procedure for such cases.^18)^

In this report, we provide 2 young patients with pancreatic trauma and MPD injury who were treated with MP with PJ reconstruction. Both cases involved delayed presentation, with surgery performed more than a day after the initial trauma. This delay resulted in significant contamination of the peritoneal cavity with pancreatic fluid, creating a challenging surgical environment. This report emphasizes that, under stable hemodynamic conditions, MP remains a viable and effective option for pancreatic trauma, even in cases of delayed presentation.

## CASE PRESENTATION

### Case 1

**Fig. 1:**
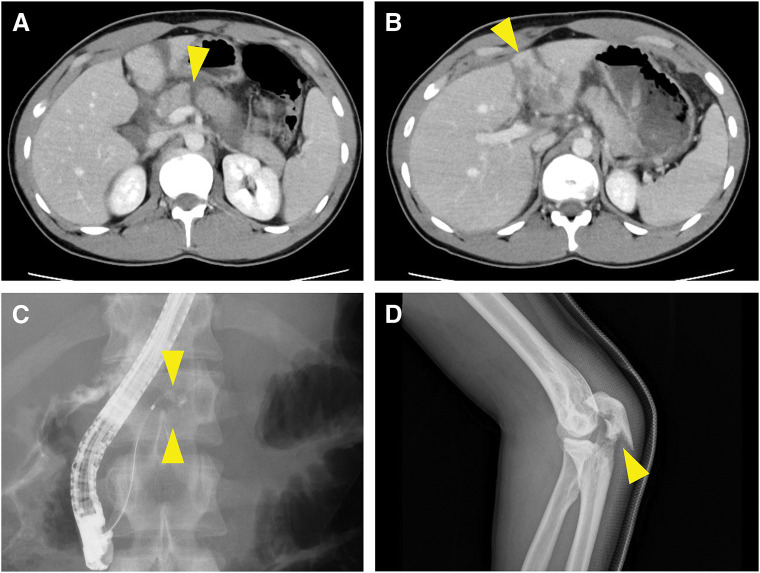
Images before surgical intervention in Case 1. (**A**) A contrast-enhanced CT scan after admission to the previous hospital revealing a laceration of the pancreatic body (arrowhead). (**B**) A contrast-enhanced CT scan after admission to the previous hospital showing a laceration and compression of the liver (arrowhead). (**C**) ERP showing the leakage of contrast agents from the MPD. (**D**) X-ray image showing a fracture of the left olecranon (arrowhead). CT, computed tomography; ERP, endoscopic retrograde pancreatography; MPD, main pancreatic duct

A 22-year-old Caucasian male (height: 180 cm; body weight: 77 kg) collided with a tree while skiing, sustaining an injury. The following day, he developed abdominal pain, and a CT scan revealed a rupture of the pancreatic body. He was diagnosed with traumatic pancreatic injury and a grade III traumatic liver injury of the left lateral segment, as classified by the American Association for the Surgery of Trauma-Organ Injury Scale (AAST-OIS) (**Fig. 1A**–**1C**).^19)^ He was transferred to our hospital 30 hours post-injury with stable hemodynamics. Laboratory tests showed elevated CRP, serum lipase, and amylase levels (**Table 1**). ERP demonstrated contrast agent leakage from the MPD (**Fig. 1C**), confirming a grade Ⅲ traumatic pancreatic injury as classified by AAST-OIS.^20)^ However, the distal pancreatic duct was not visualized. Fracture of the left olecranon was confirmed using radiography (**Fig. 1D**).

**Table 1:** Case 1: Laboratory examination on ER admission

Hematology	
White blood Cell	11600 /μL
Hemoglobin	13.4 g/dL
Platelet	215 × 10^3^/μL
Coagulation	
PT	57.5%
PT-INR	1.34 s
APTT	29.8 s
Fibrinogen	382 mg/dL
Serum chemistry	
Albumin	4.3 g/dL
Urea nitrogen	12 mg/dL
Creatinine	0.7 mg/dL
Na	131 mEq/L
K	4 mEq/L
Chloride	98 mEq/L
Total bilirubin	1.1 mg/dL
Direct bilirubin	0.1 mg/dL
AST	66 IU/mL
ALT	35 IU/mL
LDH	374 U/L
Amylase	1576 IU/L
Lipase	1812 IU/L
CRP	10.53 mg/dL
Blood gas analysis (arterial blood under 10 L/min oxygen)	
pH	7.427
PaCO_2_	36.3 mmHg
PaO_2_	95.9 mmHg
Glucose	122 mg/dL
Bicarbonate ion	0 mmol/L
Lactic acid	0.5 mmol/L

ALT, alanine aminotransferase; APTT, activated partial thromboplastin time; AST, aspartate aminotransferase; CRP, C-reactive protein; ER, emergency room; LDH, lactate dehydrogenase; PT, prothrombin time; PT-INR, PT-international normalized ratio

**Fig. 2:**
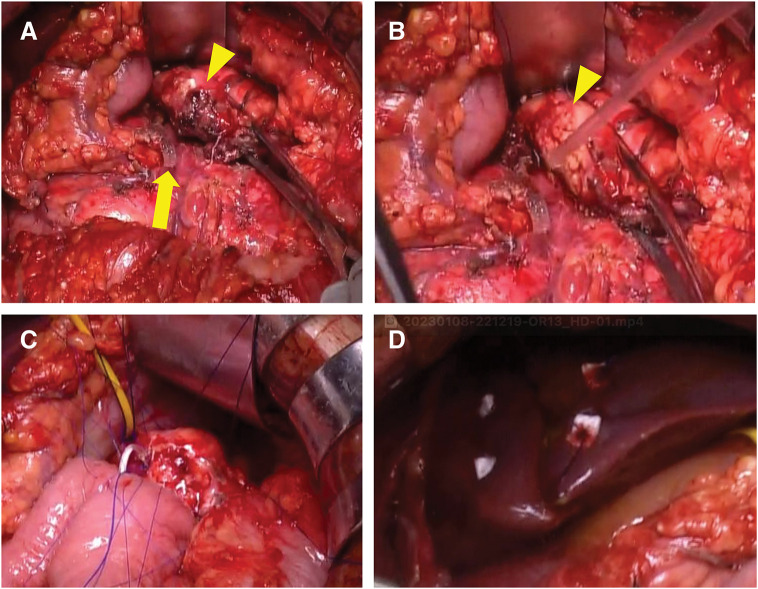
Surgical findings from Case 1. (**A**) The crushed and torn pancreatic body (arrowhead) and pancreatic head stump (arrow). (**B**) The crushed pancreatic body had been removed, and the distal resection margin was trimmed (arrowhead). (**C**) A modified Blumgart technique for pancreatojejunostomy was performed. (**D**) The lacerated liver was sutured with pledgets.

Laparotomy revealed the presence of bloody ascites mixed with bile. A liver injury in the left lateral segment was identified, with bleeding successfully controlled by packing alone, without hemodynamic deterioration. The anatomical structures of the pancreas were obscured by saponification, making initial identification challenging. Further exploration revealed that approximately half of the pancreatic neck was crushed and torn (**Fig. 2A**). The intact portion of the pancreatic neck was then exposed and dissected using a linear stapler (**Fig. 2A**). After resecting the damaged tissue and trimming the torn pancreatic body, the healthy pancreatic body parenchyma was exposed (**Fig. 2B**). A 5-Fr pancreatic duct tube was then inserted into the MPD. PJ with duct-to-mucosa anastomosis was performed using eight 5-0 PROLENE (ETHICON, Somerville, NJ, USA) sutures with a modified Blumgart technique (**Fig. 2C**), followed by Roux-en-Y reconstruction. The crushed liver area was repaired with two 2-0 PDS sutures and pledgets (**Fig. 2D**). The procedure lasted 4 hours and 34 minutes, with an estimated blood loss of 1480 mL. No clinically relevant PF developed postoperatively, and surgery for the elbow fracture was successfully performed on postoperative day 7. The patient was discharged on postoperative day 18. The patient, a traveler from Canada, is no longer under follow-up at our institution. However, as a professional skier, he returned to Japan, and by the end of last year, it was confirmed that he remained in good health, with no abdominal symptoms or complications. During the 2-year follow-up period, the patient continued his career as a professional skier without any issues or setbacks.

### Case 2

**Fig. 3:**
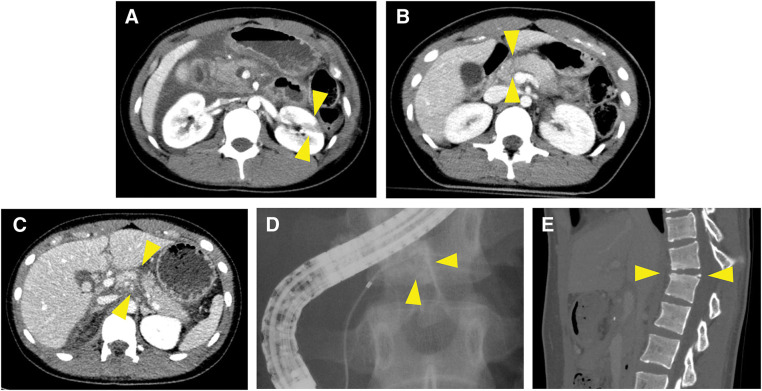
Images before the surgical intervention in Case 2. (**A**) Contrast-enhanced CT scan after admission to the previous hospital. The arrowheads mark the kidney injury (AAST-OIS grade IIIb). (**B**) Contrast-enhanced CT scan after admission to the previous hospital. The arrowheads mark a slight abnormality of the pancreatic injury. (**C**) Contrast-enhanced CT scan after admission to our hospital (3 days after injury). The arrowheads mark the location of the pancreatic injury, which is more pronounced compared to (A). (**D**) ERP revealed the leakage of contrast agents from the MPD. (**E**) Sagittal CT scan of dislocation fracture of the 11th and 12th thoracic vertebrae. AAST, American Association for the Surgery of Trauma; CT, computed tomography; ERP, endoscopic retrograde pancreatography; MPD, main pancreatic duct

**Fig. 4:**
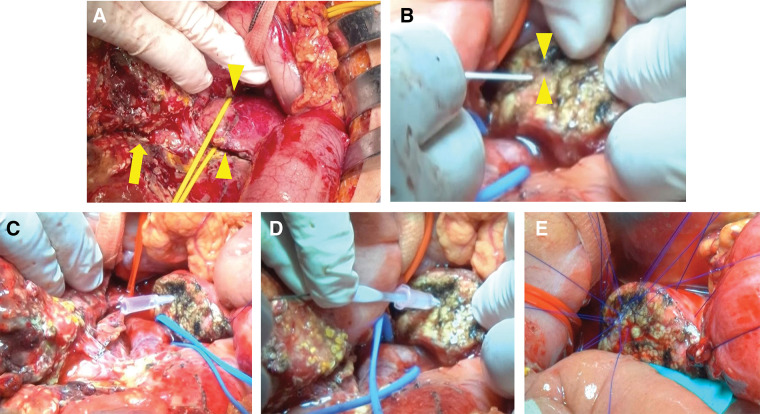
Surgical findings from Case 2. (**A**) The abdominal cavity exhibited peritonitis and adhesions with saponification (arrow). The pancreatic body was encircled by a yellow vessel loop. Pancreatic injury was observed without remarkable capsular rupture (arrowheads). (**B**) The MPD was scarcely identifiable due to its extremely small caliber. The arrowheads mark the location of the MPD. (**C**) An intravenous cannula was inserted into the MPD. (**D**) The MPD was successfully secured using an over-the-wire technique with a 0.025-inch guidewire. (**E**) Pancreatojejunostomy (duct-to-mucosa anastomosis) was performed using eight 6-0 PROLENE sutures. MPD, main pancreatic duct

A 17-year-old Asian female (height: 155 cm; body weight: 54 kg) sustained injuries in a traffic accident while seated in the rear seat of a vehicle. She was initially admitted to a nearby hospital for observation. A left kidney injury, classified as AAST-OIS grade III,^21)^ was diagnosed on the initial CT scan (**Fig. 3A**). However, the pancreatic injury was too subtle to be detected on the initial CT scan, and she was managed conservatively under observation (**Fig. 3B**). Three days later, she developed abdominal pain with a firm abdomen. CT re-evaluation revealed pancreatic body rupture and significant ascites (**Fig. 3C**), leading to her transfer to our hospital 72 hours post-injury. Laboratory findings showed elevated CRP, serum lipase, and amylase levels, similar to Case 1 (**Table 2**). Her vital signs remained stable. ERP revealed contrast extravasation from the MPD, with no visualization of the distal duct (**Fig. 3D**). Although an endoscopic pancreatic duct drainage was attempted, the drainage of the distal pancreatic duct could not be achieved. During laparotomy, the abdominal cavity showed severe peritonitis and adhesions resembling those typically observed in acute pancreatitis (**Fig. 4A**). A thorough dissection was performed to identify the anatomical structures. Although a contusion was observed beneath the capsule of the pancreatic neck, no complete capsular rupture was identified (**Fig. 4A**), which was inconsistent with the CT findings. Ultrasonographic evaluation of the MPD from the pancreatic body side demonstrated a discontinuity at the right edge of the portal vein, which was subsequently identified as the site of injury. After the dissection of the pancreatic neck with a linear stapler, the pancreatic body was transected distal to the site of MPD disruption using a scalpel. The pancreatic duct was extremely thin and challenging to identify, measuring approximately 1 mm in diameter (**Fig. 4B**), with an intravenous cannula barely able to pass through (**Fig. 4C**). A 4-Fr pancreatic duct tube was successfully placed using a 0.025-inch guidewire (**Fig. 4D**). With the pancreatic duct lumen visualized using a traction suture, duct-to-mucosa anastomosis was performed using eight 6-0 PROLENE sutures (**Fig. 4E**). The procedure was completed using the modified Blumgart technique and Roux-en-Y reconstruction, as in Case 1. This procedure lasted 7 hours and 18 minutes, with an estimated intraoperative blood loss of 1200 mL. The extended operative time was primarily owing to challenges in identifying and securing the MPD. Postoperatively, drainage therapy was required for a PF from the pancreatic head stump. However, no significant PF was observed at the PJ site, and the surrounding drains were removed early. One week after surgery for pancreatic injury, the patient developed right foot numbness. A retrospective review of the initial CT scan revealed a previously unrecognized fracture of the 11th and 12th thoracic vertebrae (**Fig. 3E**). The fracture was managed conservatively following an evaluation by the orthopedic surgeon to prioritize perioperative management of the abdominal surgery. The patient had an overall stable recovery and was discharged on postoperative day 43. The kidney injury healed uneventfully with conservative management. She subsequently underwent spinal surgery during the 2nd admission. The patient continues to undergo regular follow-up at our institution. One year postoperatively, CT imaging showed no abnormalities in the remnant pancreas or spleen. Laboratory tests indicated normal glucose tolerance and serum albumin levels, with no evidence of endocrine or exocrine insufficiency. She remains asymptomatic and leads a normal student life, having recently participated in a short-term overseas study program.

**Table 2:** Case 2: Laboratory examination on ER admission

Hematology	
White blood Cell	10100 /μL
Hemoglobin	13.9 g/dL
Platelet	200 × 10^3^/μL
Coagulation	
PT	80%
PT-INR	1.13 s
APTT	30.8 s
Fibrinogen	318 mg/dL
Serum chemistry	
Albumin	3.5 g/dL
Urea nitrogen	17 mg/dL
Creatinine	0.63 mg/dL
Na	134 mEq/L
K	4.6 mEq/L
Chloride	99 mEq/L
Total bilirubin	1.6 mg/dL
Direct bilirubin	0.2 mg/dL
AST	57 IU/mL
ALT	46 IU/mL
LDH	500 U/L
Amylase	1109 IU/L
Lipase	1673 IU/L
Creatinine	0.63 IU/L
CRP	22.13 mg/dL
Blood gas analysis (arterial blood under 10 L/min oxygen)	
pH	7.428
PaCO_2_	40.3 mmHg
PaO_2_	65.8 mmHg
Glucose	140 mg/dL
Base excess	2.2 mmol/L
Lactic acid	0.9 mmol/L

ALT, alanine aminotransferase; APTT, activated partial thromboplastin time; AST, aspartate aminotransferase; CRP, C-reactive protein; ER, emergency room; LDH, lactate dehydrogenase; PT, prothrombin time; PT-INR, PT-international normalized ratio

## DISCUSSION

Blunt pancreatic injury is uncommon, and the optimal management strategy, including the choice of surgical approach, remains undefined.^22)^ MP with Roux-en-Y PJ, also known as the Letton–Wilson procedure, is occasionally performed to preserve organ function in cases of pancreatic body injuries with MPD disruption.^11,12)^ However, its applicability in the context of trauma remains unclear. In this report, we present 2 young patients with pancreatic trauma and MPD injury who were successfully treated with this procedure. Despite being hemodynamically stable, both patients presented with delayed diagnosis, 1–3 days post-injury, leading to severe erosion of the surrounding pancreatic tissue (**Fig. 4A**). The surgical approach was technically demanding, particularly the duct-to-mucosa suturing, due to the extremely small MPD and severe inflammatory changes in the pancreatic tissue (**Figs. 2C** and **4E**). Nevertheless, this case series highlights the feasibility of MP with PJ as a life-saving and organ-preserving option, even in the setting of delayed presentation.

MP was 1st described in 1910^23)^ and has traditionally been used for benign or low-grade malignant pancreatic tumors. It was later introduced as a potential surgical option for pancreatic trauma.^11)^ Unlike DP, MP preserves both the pancreatic parenchyma and the spleen, potentially reducing postoperative pancreatic dysfunction.^13)^ A meta-analysis of 94 studies on MP provides valuable insights into the complication rates and recovery outcomes.^24)^ It found that MP was associated with higher postoperative morbidity and a greater incidence of PF compared to DP. However, MP carries a lower risk of endocrine and exocrine insufficiency.^24)^ Furthermore, a 2024 international retrospective multicenter study and a 2020 meta-analysis reported new-onset diabetes mellitus rates of 11% following MP^25)^ and 29% following DP,^26)^ respectively. Additionally, splenic preservation is associated with a reduced long-term risk of overwhelming post-splenectomy infection, particularly in young patients, offering significant immunological benefits.^27)^ Neither the Western Trauma Association (WTA) nor the Eastern Association for the Surgery of Trauma (EAST) guidelines specifically address MP, and the procedure is not mentioned in either of the 2 guidelines.^17,28)^ The WTA guidelines propose a decision-making algorithm for blunt pancreatic trauma, recommending pancreatectomy with or without splenectomy for high-grade injuries located to the left of the superior mesenteric vein.^28)^ Similarly, the EAST guidelines discuss the routine use of splenectomy with DP; however, owing to a lack of direct comparative studies, no definitive recommendation is provided. Additionally, the studies found no significant difference in mortality between patients who underwent splenectomy and those who did not. MP carries a higher short-term risk of complications, particularly PF, due to the need for pancreatic reconstruction.^29)^ This risk must be weighed against its long-term advantages, particularly for young patients, for whom organ preservation may provide significant metabolic and endocrine benefits.

While nonoperative management using pancreatic duct stenting is a less invasive approach for pancreatic trauma, it is contraindicated in cases of complete injury with disrupted MPD continuity.^7)^ This approach is only applicable under specific conditions, including isolated MPD injury, hemodynamic stability, localized peritonitis, absence of axial displacement of the MPD, no evidence of extensive contrast leakage on ERP, and clear visualization of the distal MPD.^30)^ In the cases presented in this study, the distal MPD could not be visualized, and the presence of multiple associated injuries, particularly in Case 1, excluded the use of a nonoperative strategy. Given the potential for treatment failure, the EAST guidelines currently recommend operative management for grade III pancreatic injuries.^17)^

The reconstruction method is a critical consideration in MP for MPD injuries. Pancreatogastrostomy (PG) may offer certain advantages, including a potentially lower incidence of postoperative PF.^31)^ In particular, the dunking technique can be a viable option in cases with a small, fragile MPD under severe inflammation, as observed in Case 2.^32)^ However, one study reported that long-term exocrine function following PG may be inferior to that achieved with PJ.^33)^ Furthermore, the dunking method requires adequate mobilization of the pancreatic stump, which can be technically challenging in the setting of severe inflammation. Therefore, we consider PJ to be the most reliable option for MP reconstruction in the context of pancreatic trauma, particularly in young patients. A previous report showed that the use of a standardized anastomotic technique can reduce the overall incidence of postoperative complications related to pancreatoenterostomy.^34)^ At our institution, we routinely perform PJ using a modified Blumgart technique for elective pancreatectomy. Nonetheless, PG or the dunking method may still be considered in select trauma cases when performed by experienced surgical teams.

A delayed diagnosis and surgical intervention, as seen in our cases, have been associated with increased morbidity and mortality in MPD injuries.^4)^ Delayed presentation often leads to saponification and tissue liquefaction around the pancreas, mimicking severe pancreatitis and creating a highly challenging surgical environment. These conditions complicate vascular dissection, anastomosis, and overall operative safety.^10)^ In our cases, surgery was performed under similarly severe inflammatory conditions, necessitating several technical modifications, including precise pancreatic trimming and secure identification of the small pancreatic duct (**Figs. 2B** and **4C**). Given these challenges, performing an organ-preserving pancreatic resection in the context of delayed presentation is only feasible when specific criteria are met. A high-volume hepatopancreatobiliary (HPB) surgical team experienced in complex pancreatic surgery is essential to ensure safe execution and optimal outcomes.^35)^ Additionally, patient stability is a critical prerequisite, as the procedure is time-consuming and technically demanding. Consequently, the patient's age should be considered when determining eligibility, as older age is a significant risk factor for mortality in pancreatic trauma.^36)^ Furthermore, in cases of polytrauma, extra caution is required, as function-preserving pancreatic resection is generally not considered an ideal approach. In Case 1, hemostatic control of the liver injury was fortunately achieved without difficulty. However, had the management of the liver injury been more complex, damage control surgery or a simpler approach, such as DP, would have been necessary. Thus, it is essential to conduct a meticulous, phase-based assessment of the feasibility at both the preoperative and intraoperative stages when performing MP for pancreatic trauma with delayed presentation.

**Fig. 5:**
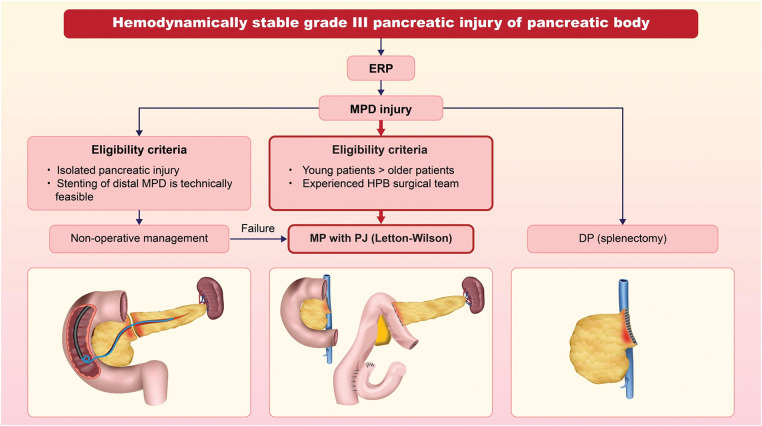
Decision-making algorithm for the treatment of main pancreatic duct injury in the pancreatic body. This algorithm applies to hemodynamically stable patients with grade III pancreatic injury involving the pancreatic body. The initial assessment includes ERP, to evaluate the feasibility of nonoperative management via stenting of the distal MPD. If stenting is not feasible, surgical management is indicated. DP with splenectomy is the standard option, while MP with pancreatojejunostomy may be considered in young patients, provided the procedure is performed by an experienced surgical team. DP, distal pancreatectomy; ERP, endoscopic retrograde pancreatography; MP, middle pancreatectomy; MPD, main pancreatic duct

Overall, while MP with PJ can be a technically feasible option for pancreatic trauma with delayed presentation, its indication should be carefully assessed.^37)^ A decision-making algorithm for the treatment of MPD injury in the pancreas body is shown in **Fig. 5**. Given the high technical demands and potential complications, it should be reserved for selected patients in high-volume centers with experienced HPB surgical teams. In appropriately selected cases, this approach may offer significant long-term benefits, particularly for young patients, by preserving both pancreatic and splenic functions. This case report serves as a valuable educational resource for HPB or trauma surgeons, providing practical guidance on decision-making and surgical execution in similar scenarios.

## CONCLUSIONS

We present 2 cases of MP with Roux-en Y PJ for MPD injuries caused by blunt trauma. Despite the complexity of the surgical procedure, it can be a valuable option for young patients in stable hemodynamic conditions.

## ACKNOWLEDGMENTS

We would like to thank Editage (www.editage.jp) for English-language editing and for the design and creation of the figures.

## DECLARATIONS

### Funding

The authors declare that they have no financial interests to disclose.

### Authors’ contributions

YI, ST, TNo, YE, MW, KT, AM, YN, TA, TNa, and SH administered clinical treatments, performed surgical procedures, and managed perioperative care.

YI and ST drafted the manuscript, with ST and SH providing expertise and feedback.

All authors reviewed and approved the final manuscript.

### Availability of data and materials

Not applicable.

### Ethics approval and consent to participate

Not applicable.

### Consent for publication

Consent to publish was obtained from the patients involved in this study.

### Competing interests

The authors declare that they have no competing interests.

## References

[1] O’Reilly DA Bouamra O Kausar A The epidemiology of and outcome from pancreatoduodenal trauma in the UK, 1989–2013. Ann R Coll Surg Engl 2015; 97: 125–30. 25723689 10.1308/003588414X14055925060712 PMC4473389

[2] Ando Y Okano K Yasumatsu H Current status and management of pancreatic trauma with main pancreatic duct injury: A multicenter nationwide survey in Japan. J Hepatobiliary Pancreat Sci 2021; 28: 183–91. 33280257 10.1002/jhbp.877 PMC7986433

[3] Heuer M Hussmann B Lefering R Pancreatic injury in 284 patients with severe abdominal trauma: outcome, course, and treatment algorithm. Langenbecks Arch Surg 2011; 396: 1067–76. 21847623 10.1007/s00423-011-0836-1

[4] Oláh A Issekutz A Haulik L Pancreatic transection from blunt abdominal trauma: early versus delayed diagnosis and surgical management. Dig Surg 2003; 20: 408–14. 12900531 10.1159/000072708

[5] Bradley EL 3rd Young PR Jr. Chang MC Diagnosis and initial management of blunt pancreatic trauma: guidelines from a multiinstitutional review. Ann Surg 1998; 227: 861–9. 9637549 10.1097/00000658-199806000-00009 PMC1191392

[6] Iacono C Zicari M Conci S Management of pancreatic trauma: a pancreatic surgeon’s point of view. Pancreatology 2016; 16: 302–8. 26764528 10.1016/j.pan.2015.12.004

[7] Lin BC Wong YC Chen RJ Major pancreatic duct continuity is the crucial determinant in the management of blunt pancreatic injury: a pancreatographic classification. Surg Endosc 2017; 31: 4201–10. 28281124 10.1007/s00464-017-5478-0

[8] Sugimoto M Takahashi S Kojima M In patients with a soft pancreas, a thick parenchyma, a small duct, and fatty infiltration are significant risks for pancreatic fistula after pancreaticoduodenectomy. J Gastrointest Surg 2017; 21: 846–54. 28101719 10.1007/s11605-017-3356-7

[9] Rozich NS Morris KT Garwe T Blame it on the injury: trauma is a risk factor for pancreatic fistula following distal pancreatectomy compared with elective resection. J Trauma Acute Care Surg 2019; 87: 1289–300. 31765347 10.1097/TA.0000000000002495 PMC7799849

[10] Lin BC Chen RJ Hwang TL. Spleen-preserving versus spleen-sacrificing distal pancreatectomy in adults with blunt major pancreatic injury. BJS Open 2018; 2: 426–32. 30511043 10.1002/bjs5.89 PMC6253790

[11] Letton AH Wilson JP. Traumatic severance of pancreas treated by Roux-Y anastomosis. Surg Gynecol Obstet 1959; 109: 473–8. 14416087

[12] Somasekar R Krishna PS Kesavan B A pragmatic approach to pancreatic trauma: A single-center experience from a tertiary care center. Cureus 2022; 14: e24793. 35677008 10.7759/cureus.24793 PMC9168422

[13] Borkon MJ Morrow SE Koehler EA Operative intervention for complete pancreatic transection in children sustaining blunt abdominal trauma: revisiting an organ salvage technique. Am Surg 2011; 77: 612–20. 21679597

[14] Johnson MA Rajendran S Balachandar TG Central pancreatectomy for benign pancreatic pathology/trauma: is it a reasonable pancreas-preserving conservative surgical strategy alternative to standard major pancreatic resection? ANZ J Surg 2006; 76: 987–95. 17054548 10.1111/j.1445-2197.2006.03916.x

[15] Hirono S Tani M Kawai M A central pancreatectomy for benign or low-grade malignant neoplasms. J Gastrointest Surg 2009; 13: 1659–65. 19488821 10.1007/s11605-009-0934-3

[16] Biffl WL Moore EE Croce M Western Trauma Association critical decisions in trauma: management of pancreatic injuries. J Trauma Acute Care Surg 2013; 75: 941–6. 24256664 10.1097/TA.0b013e3182a96572

[17] Ho VP Patel NJ Bokhari F Management of adult pancreatic injuries: a practice management guideline from the Eastern Association for the Surgery of Trauma. J Trauma Acute Care Surg 2017; 82: 185–99. 27787438 10.1097/TA.0000000000001300

[18] Søreide K Weiser TG Parks RW. Clinical update on management of pancreatic trauma. HPB (Oxford) 2018; 20: 1099–108. 30005994 10.1016/j.hpb.2018.05.009

[19] Moore EE Cogbill TH Jurkovich GJ Organ injury scaling: spleen and liver (1994 Revision). J Trauma 1995; 38: 323–4. 7897707 10.1097/00005373-199503000-00001

[20] Moore EE Cogbill TH Malangoni MA Organ injury scaling, 11: Pancreas, duodenum, small bowel, colon, and rectum. J Trauma 1990; 30:1427–9. 2231822

[21] Patel P Duttaroy D Kacheriwala S. Management of renal injuries in blunt abdominal trauma. J Res Med Dent Sci 2014; 2: 38–42.

[22] Shibahashi K Sugiyama K Kuwahara Y Epidemiological state, predictive model for mortality, and optimal management strategy for pancreatic injury: A multicentre nationwide cohort study. Injury 2020; 51: 59–65. 31431334 10.1016/j.injury.2019.08.009

[23] Finney JM. VII. Resection of the pancreas: report of a case. Ann Surg 1910; 51: 818–29. 17862541 10.1097/00000658-191006000-00007 PMC1406055

[24] Iacono C Verlato G Ruzzenente A Systematic review of central pancreatectomy and meta-analysis of central versus distal pancreatectomy. Br J Surg 2013; 100: 873–85. 23640664 10.1002/bjs.9136

[25] van Bodegraven EA Lof S Jones L Tailoring the use of central pancreatectomy through prediction models for major morbidity and postoperative diabetes: International retrospective multicenter study. Ann Surg 2024; 280: 993–8. 38073561 10.1097/SLA.0000000000006157 PMC11542965

[26] Yu J Sun R Han X New-onset diabetes mellitus after distal pancreatectomy: A systematic review and meta-analysis. J Laparoendosc Adv Surg Tech A 2020; 30: 1215–22. 32559393 10.1089/lap.2020.0090

[27] Okabayashi T Hanazaki K. Overwhelming postsplenectomy infection syndrome in adults—a clinically preventable disease. World J Gastroenterol 2008; 14: 176–9. 18186551 10.3748/wjg.14.176 PMC2675110

[28] Moren AM Biffl WL Ball CG Blunt pancreatic trauma: A Western Trauma Association critical decisions algorithm. J Trauma Acute Care Surg 2023; 94: 455–60. 36397206 10.1097/TA.0000000000003794

[29] Paiella S De Pastena M Faustini F Central pancreatectomy for benign or low-grade malignant pancreatic lesions—A single-center retrospective analysis of 116 cases. Eur J Surg Oncol 2019; 45: 788–92. 30527222 10.1016/j.ejso.2018.11.021

[30] Bhasin DK Rana SS Rawal P. Endoscopic retrograde pancreatography in pancreatic trauma: need to break the mental barrier. J Gastroenterol Hepatol 2009; 24: 720–8. 19383077 10.1111/j.1440-1746.2009.05809.x

[31] Topal B Fieuws S Aerts R Pancreaticojejunostomy versus pancreaticogastrostomy reconstruction after pancreaticoduodenectomy for pancreatic or periampullary tumours: a multicentre randomised trial. Lancet Oncol 2013; 14: 655–62. 23643139 10.1016/S1470-2045(13)70126-8

[32] Giulianotti PC Gonzalez-Heredia R Esposito S Trans-gastric pancreaticogastrostomy reconstruction after pylorus-preserving robotic Whipple: a proposal for a standardized technique. Surg Endosc 2018; 32: 2169–74. 29247370 10.1007/s00464-017-5916-z

[33] Hirono S Murakami Y Tani M Identification of risk factors for pancreatic exocrine insufficiency after pancreaticoduodenectomy using a ^13^C-labeled mixed triglyceride breath test. World J Surg 2015; 39:516–25. 25318451 10.1007/s00268-014-2832-4

[34] Shrikhande SV Sivasanker M Vollmer CM Pancreatic anastomosis after pancreatoduodenectomy: A position statement by the International Study Group of Pancreatic Surgery (ISGPS). Surgery 2017; 161: 1221–34. 28027816 10.1016/j.surg.2016.11.021

[35] Hata T Motoi F Ishida M Effect of hospital volume on surgical outcomes after pancreaticoduodenectomy: A systematic review and meta-analysis. Ann Surg 2016; 263:664–72. 26636243 10.1097/SLA.0000000000001437

[36] Krige JE Kotze UK Setshedi M Prognostic factors, morbidity and mortality in pancreatic trauma: a critical appraisal of 432 consecutive patients treated at a Level 1 Trauma Centre. Injury 2015; 46: 830–6. 25724398 10.1016/j.injury.2015.01.032

[37] Lin BC Chen RJ Hwang TL. Lessons learned from isolated blunt major pancreatic injury: Surgical experience in one trauma centre. Injury 2019; 50: 1522–8. 31164222 10.1016/j.injury.2019.05.027

